# Glutaminase and MMP-9 Downregulation in Cortex and Hippocampus of LPA_1_ Receptor Null Mice Correlate with Altered Dendritic Spine Plasticity

**DOI:** 10.3389/fnmol.2017.00278

**Published:** 2017-09-05

**Authors:** Ana Peñalver, José A. Campos-Sandoval, Eduardo Blanco, Carolina Cardona, Laura Castilla, Mercedes Martín-Rufián, Guillermo Estivill-Torrús, Raquel Sánchez-Varo, Francisco J. Alonso, Mercedes Pérez-Hernández, María I. Colado, Antonia Gutiérrez, Fernando Rodríguez de Fonseca, Javier Márquez

**Affiliations:** ^1^Canceromics Laboratory, Departamento de Biología Molecular y Bioquímica, Facultad de Ciencias, Universidad de Málaga, Campus de Teatinos Málaga, Spain; ^2^Unidad de Gestión Clínica de Salud Mental, Instituto de Investigación Biomédica de Málaga (IBIMA), Hospital Regional Universitario de Málaga Málaga, Spain; ^3^Unidad de Gestión Clínica de Neurociencias, Instituto de Investigación Biomédica de Málaga (IBIMA), Hospital Regional Universitario de Málaga Málaga, Spain; ^4^Departamento de Biología Celular, Genética y Fisiología, Facultad de Ciencias, Instituto de Investigación Biomédica de Málaga (IBIMA), Centro de Investigación Biomédica en Red sobre Enfermedades Neurodegenerativas (CIBERNED), Universidad de Málaga, Campus de Teatinos Málaga, Spain; ^5^Departamento de Farmacología, Facultad de Medicina, Universidad Complutense, Instituto de Investigación Sanitaria Hospital 12 de Octubre Madrid, Spain

**Keywords:** lysophosphatidic acid, glutaminase, glutamate, matrix metalloproteinases, rodent knockout model, synaptic plasticity

## Abstract

Lysophosphatidic acid (LPA) is an extracellular lipid mediator that regulates nervous system development and functions acting through G protein-coupled receptors (GPCRs). Here we explore the crosstalk between LPA_1_ receptor and glutamatergic transmission by examining expression of glutaminase (GA) isoforms in different brain areas isolated from wild-type (WT) and KOLPA_1_ mice. Silencing of LPA_1_ receptor induced a severe down-regulation of Gls-encoded long glutaminase protein variant (KGA) (glutaminase gene encoding the kidney-type isoforms, GLS) protein expression in several brain regions, particularly in brain cortex and hippocampus. Immunohistochemical assessment of protein levels for the second type of glutaminase (GA) isoform, glutaminase gene encoding the liver-type isoforms (GLS2), did not detect substantial differences with regard to WT animals. The regional mRNA levels of GLS were determined by real time RT-PCR and did not show significant variations, except for prefrontal and motor cortex values which clearly diminished in KO mice. Total GA activity was also significantly reduced in prefrontal and motor cortex, but remained essentially unchanged in the hippocampus and rest of brain regions examined, suggesting activation of genetic compensatory mechanisms and/or post-translational modifications to compensate for KGA protein deficit. Remarkably, Golgi staining of hippocampal regions showed an altered morphology of glutamatergic pyramidal cells dendritic spines towards a less mature filopodia-like phenotype, as compared with WT littermates. This structural change correlated with a strong decrease of active matrix-metalloproteinase (MMP) 9 in cerebral cortex and hippocampus of KOLPA_1_ mice. Taken together, these results demonstrate that LPA signaling through LPA^1^ influence expression of the main isoenzyme of glutamate biosynthesis with strong repercussions on dendritic spines maturation, which may partially explain the cognitive and learning defects previously reported for this colony of KOLPA^1^ mice.

## Introduction

Lysophosphatidic acid (LPA; 1 or 2-acyl-*sn*-glycero-3-phosphate) is a naturally occurring lysophospholipid derived from cell membranes that belongs to the growing list of bioactive lipids. It may act as an intercellular signaling molecule after recognition by G protein-coupled receptor(s) (GPCRs). In mammals, LPA is enriched in brain tissue, produced by platelets and cells from the nervous system and is also found in serum (Choi and Chun, 2013; Yung et al., 2015). Six types of LPA receptors (LPARs) have been identified so far named LPA_1_-LPA_6_, with different expression patterns and involved in different functions (Chun et al., 2010; Mutoh et al., 2012; Yung et al., 2014).

Multiple cellular responses have been reported for this bioactive natural lipid in the central nervous system (CNS) as well as in neural cell lines. LPA elicited sharp changes in cell’s shape and morphology, growth cone collapse and neurite retraction in many neuronal cell lines through activation of the small GTPase Rho which in turn contracts cytoskeleton (Ye et al., 2002; Choi et al., 2010; Fukushima et al., 2011). Furthermore, LPA also affect cell motility in glioma cell lines through Rho/Rho-associated kinases (ROKC) signaling cascades, which may contribute to their invasive phenotype *in vivo* (Manning et al., 2000). In cultured astrocytes, LPA induces a plethora of responses including proliferation and inhibition of glutamate uptake (Steiner et al., 2002; Shano et al., 2008). Of note, LPA-primed astrocytes promote neuronal differentiation of cerebral cortical progenitors and developing cortical neurons, which showed increases in arborization and neurite outgrowth (Spohr et al., 2008, 2011).

Although many types of LPARs have been detected in brain, LPA_1_ is abundantly expressed and considered the most prevalent receptor type in both embryonic and adult brains of humans and mice (Hecht et al., 1996; Choi et al., 2010; Mutoh et al., 2012). Genetic silencing of LPA_1_ in mice causes a reduced ventricular zone along with loss of cortical layer cellularity (Estivill-Torrús et al., 2008), reduces neurogenesis in the dentate gyrus (DG; Matas-Rico et al., 2008), and shows altered neurotransmitter homeostasis (Musazzi et al., 2011; Blanco et al., 2012a) which were previously related to psychiatric diseases (Harrison et al., 2003; Roberts et al., 2005). Behavioral studies with LPA_1_ deficient mice reported deficiencies in spatial memory retention and abnormal use of searching orientation strategies (Santin et al., 2009), defective working and reference memory independently of exploratory and emotional impairments attributed to hippocampal malfunction (Castilla-Ortega et al., 2010). Lastly, mice lacking LPA_1_ receptor exhibit an endophenotype for alcohol preference associated with glutamate receptor alterations in the prefrontal cortex (PFC), including a decrease in their relative mRNA levels of Gls-encoded long glutaminase protein variant (KGA; Castilla-Ortega et al., 2016).

Studies dealing with KO models of LPARs are giving new insights into the role of LPA in developmental and differentiation processes of neurons and glial cells and their cross-talk in the tripartite synapsis; however, little is known about how LPA signaling regulates synaptic function and neurotransmission (Ye et al., 2002; García-Morales et al., 2015). LPA may modify N-methyl-D-aspartate (NMDA) receptor functions in hippocampal neurons (Lu et al., 1999) as well as calcium intracellular levels (Holtsberg et al., 1997). Furthermore, LPA inhibited Na^+^, K^+^-ATPase activity in rat cortical synaptosomes (Nishikawa et al., 1989) and was related with synaptic vesicle formation (Schmidt et al., 1999). In addition, LPA through LPA_1_ receptor has been proposed as a potential candidate to control short-term synaptic plasticity both in excitatory and inhibitory synapses, but using different mechanisms: decreasing the synaptic vesicle pool and reducing the number of postsynaptic receptors, respectively (García-Morales et al., 2015). The aim of this study was to elucidate further relationships between LPA signaling and synaptic plasticity of glutamatergic excitatory transmission using KOLPA_1_ mice as a model. We found marked regional down-regulations of the main glutaminase (GA; EC 3.5.1.2) isoform (KGA) involved in the synthesis of neurotransmitter glutamate, altered dendritic spines morphology at the glutamatergic pyramidal cells of the hippocampus and a decrease in MMP-9 activity and expression, an enzyme crucially required for dendritic spine maturation and remodeling. Altogether, the results point to important implications of LPA in synaptic excitatory plasticity which may contribute to the cognitive and memory deficits shown by LPA_1_-deficient mice.

## Materials and Methods

### Animals

The generation and characterization of maLPA_1_-null mice has been described previously (Estivill-Torrús et al., 2008). An LPA_1_-null mouse colony, termed maLPA_1_ after the Málaga variant of the LPA_1_ knockout, was spontaneously derived during the original colony (Contos et al., 2000) expansion by crossing heterozygous (HT) foundation parents (maintained in the original hybrid C57BL/6J6x1291/SvJ background). Intercrosses were performed with these mice and were subsequently backcrossed for 20 generations with mice generated within this mixed background. MaLPA_1_-null mice carrying the LPA_1_ deletion were born at the expected Mendelian ratio and survived to adulthood. Targeted disruption of the *Lpa1* gene was confirmed by genotyping (Contos et al., 2000), and immunochemistry confirmed the absence of LPA_1_ protein expression. Three month-old male littermates from wild-type (WT), heterozygous and malLPA_1_-null homozygous (KOLPA_1_) genotypes were employed for immunohistochemistry. In all other experiments, WT and KOLPA_1_ genotypes were employed. Mice were singly housed on a 12-h light/dark cycle (lights on at 07:00 a.m.). Water and food were provided *ad libitum*. All animal experiments were performed in accordance with the animal research regulations (RD53/2013 and 2010/63/UE) from Spain and European Union, and with the approval of the Committees of Animal Research from the University of Malaga (Spain).

### Immunohistochemistry of Glutaminase Isoforms

For immunohistochemical studies animals (*n* = 8 mice per genotype) were deeply anesthetized with sodium pentobarbital (60 mg/kg) and perfused transcardially with 0.1 M phosphate buffer (PB) saline (PBS), pH 7.4, followed by fixative solution (4% paraformaldehyde, 75 mM lysine and 10 mM sodium metaperiodate in 0.1 M PB, pH 7.4). After overnight postfixation in the same fixative at 4°C, brains were cryoprotected (30% sucrose), sectioned (40 μm) on a freezing microtome and serially collected (six series for each brain, with a separation of 240 μm between consecutive sections) in wells containing cold PBS and 0.02% sodim azide. The rabbit polyclonal isoform-specific antibodies anti-KGA and anti-GAB (glutaminase gene encoding the liver-type isoforms (G*ls2*)-encoded long GA isoform) were generated and characterized in our laboratory (Olalla et al., 2002, 2008; Campos et al., 2003; Cardona et al., 2015). Briefly, free-floating sections, after blocking the endogenous peroxidase activity (3% H_2_O_2_/10% methanol in 0.1 M PBS pH 7.4 for 20 min) and avidin, biotin, and biotin-binding proteins for 30 min with avidin-biotin blocking Kit (Vector Labs, United Kingdom), were incubated with the affinity-purified rabbit polyclonal anti-KGA antibody (1:1000) or rabbit polyclonal anti-GAB antibody (1:500 dilution) for 48 h at room temperature. The tissue-bound primary antibody was then detected by incubating with biotinylated anti-rabbit IgG (Vector Labs, United Kingdom) for 1 h at room temperature (1:500 dilution) and then with ExtrAvidin-peroxidase conjugate (Sigma-Aldrich, Spain) for 1 h at room temperature (1:2000 dilution). Immunoreaction product was visualized with 0.05% 3-3′-diaminobenzidine tetrahydrochloride (DAB; Sigma-Aldrich, Spain), 0.03% nickel ammonium sulfate, and 0.01% H_2_O_2_ in PBS. Sections were then mounted onto gelatin-coated slides, dehydrated in graded ethanol, cleared in xylene and coverslipped with DPX mounting medium (VWR BDH Prolabo, VWR International). Immunostaining was observed under a Nikon Eclipse 80i microscope and images acquired with a Nikon DS-5M high-resolution digital camera. All immunohistochemical studies by light microscopy were tested using negative controls (omitting the primary antisera) and no immunoreaction products were detected in any case.

### Golgi Staining

Golgi staining with FD Rapid GolgiStain™ kit (Golgi-Cox technique; FD Neurotechnologies) was performed to detect morphological alterations in neuronal dendrites and dendritic spines. The kit was used following the provider instructions. Briefly, adult male mice from both genotypes, WT and KOLPA_1_ (*n* = 5 per group), were deeply anesthetized with sodium pentobarbital (60 mg/kg) and brains were immediately removed. Tissues were rinsed in distilled water and immersed in the fixative solution (A + B) during 2 weeks in the dark. Afterwards, brains were transferred to solution C for 4 days. Before sectioning on a freezing microtome (Leica, CM 1325), tissue was rapidly frozen in isopentane precooled to −70°C. Sections of 150 μm were mounted with solution C on gelatine-coated slides and air-dried at RT. Then, the kit staining procedure was followed, and slides were dehydrated in graded ethanol, cleared in xylene and coverslipped with DPX (BDH) mounting medium.

Microphotographs of basal pyramidal dendrites in CA1 stratum oriens from hippocampus were acquired using a 63× oil-immersion objective of a SP5 II microscope (Leica) with a 405 Diode, UV Laser. Series of Z-stacks of dendrites were taken with 0.3 μm spacing. LAS AF Lite (Leica) software was used for quantification and morphometric analysis. For quantitative analysis, we assessed nonprimary dendritic segments, using the following criteria: (1) the quality of Golgi staining; (2) the relative isolation of the dendrites; (3) we selected only the spines that protruded in the transverse direction and were clearly distinguished. Spine density (number of spines per 10 μm), as well as dendrite diameter at several points of each counted segment, were measured. Other parameters such as spine morphology and length were also evaluated. Protrusions from dendrites were classified based on predefined parameters as mushroom spines, stubby spines, thin spines, or filopodia (Ghani et al., 2017). A total of 527 dendritic spines (283 spines for WT and 244 for KOLPA_1_ mice) were analyzed. Finally, mushroom head size was assessed, defining the head width (μm) as the diameter of the largest spine section that was perpendicular to a virtual axis of the spine trajectory (Ruszczycki et al., 2012).

### Real-Time Quantitative RT-PCR

For qRT-PCR analyses (and enzymatic assays), animals were sacrificed by decapitation. Brains were quickly removed, frozen (−80°C) and dissected in coronal brain slices (2 mm thickness) with razor blades in a mouse brain slicer matrix (Zivic Instruments). The discrete brain regions (striatum, hippocampus, PFC and cerebellum) were picked up by free hand dissection using a scalpel. These brain regions were identified according to Mouse Brain in Stereotaxic Coordinates (Paxinos and Franklin, 2001) as described previously (Blanco et al., 2012a): striatum from the Bregma 1.54 mm to −0.46 mm, hippocampus from the Bregma −1.22 mm to −3.52 mm, PFC from the Bregma 2.46 mm to 1.34 mm and cerebellum from the Bregma −5.52 mm to −7.80 mm.

Total RNA from brain regions was isolated according to manufacturer’s specifications (AllPrep DNA/RNA/Protein Mini Kit, Qiagen). Total RNA (1 μg) were reverse transcribed using the Quantitec Reverse Transcription kit (Qiagen) in a 20 μl reaction, according to manufacturer’s instructions. RNA samples were tested for genomic DNA contamination by including reverse transcriptase (RT) omitted controls, and for reagents and aerosol contamination by including two non-template controls. Real time PCR was carried out with the CFX thermocycler (Bio-Rad) as described elsewhere (Martín-Rufián et al., 2012). GLS transcripts were amplified using mouse GLS-specific primers: GLS forward (5′-GCGAGGGCAAGGAGATGGTG-3′) and GLS reverse (5′-CTCTTTCAACCTGGGATCAGA TGTTC-3′), as described previously (Martín-Rufián et al., 2012). Results were evaluated using CFX Bio-Rad Analysis Software. Amplicon size and the absence of non-specific products were confirmed by agarose gel electrophoresis. For absolute quantification of GA transcripts, PCR transcript-specific amplicons were prepared as standards. PCR products were analyzed by 2% agarose gel electrophoresis and purified (Illustra™ GFXTMPCR DNA and Gel Band Purification Kit, GE Healthcare). The standard curve was created by plotting the threshold cycle (Ct) corresponding to each standard, vs. the value of their corresponding log of starting quantity (DNA copy number). GLS transcripts were expressed as numbers of molecules (± SEM) of mRNA per ng of total RNA. Five animals of each genotype (WT and KOLPA_1_) were employed and independent experiments were performed in triplicate.

### Glutaminase Activity Assay

Brain regions were isolated as described above. The discrete brain regions from dissections were resuspended in TES buffer (25 mM Tris–HCl, 0.2 mM EDTA, 0.33 M sucrose, pH 8.0) containing the complete protease inhibitor cocktail (Roche), homogenized with a Pestle motor mixer (VWR) and solubilized with TX-100 at a final concentration of 1% (v/v) for 1 h at 4°C. After centrifugation at 100,000× *g* for 30 min at 4°C, the supernatants were divided into aliquots and kept at −80°C until analysis. The protein content in each sample was determined by the Bradford method. GA activity was assayed by measuring the ammonia produced in the catalytic reaction as described by Heini et al. (1987). Briefly, samples of 25 μL were added to 35 μL of a mixture of 100 mM potassium phosphate, 171 mM L-Gln and 1.5 mM NH_4_Cl, pH 8.0 and incubated for 1 h at 37°C. The reaction was terminated by adding 10 μL of trichloroacetic acid (TCA) 10% (w/v), kept on ice for 15 min and centrifuged at 12,000× *g*. Aliquots of 5 μL were then mixed with 150 μL of o-phthalaldehyde/mercaptoethanol reagent (10 mL 0.2 M potassium phosphate, pH 7.4, 0.56 mL of 72 mM mercaptoethanol in ethanol and 0.56 mL of 186 mM o-phthalaldehyde in ethanol). The samples were kept at room temperature in the dark and their absorbance measured at 410 nm after 45 min together with an NH_4_Cl standard. For blanks, the samples and substrate solution were incubated separately and mixed after the addition of TCA.

### Gel Zymography of MMP-9 and MMP-2 Activity

Brain regions were homogenized in nonyl phenoxypolyethoxylethanol (NP-40, Thermo Fisher) lysis buffer (150 mM NaCl, 50 mM Tris-HCl, 1% NP-40, pH 7.4) supplemented by 5% protease inhibitor cocktail (Sigma-Aldrich, Spain) and 1% phosphatase inhibitor cocktail-2 (Sigma-Aldrich, Spain). Protein concentrations were measured by DC Protein Assay kit (Bio-Rad, Spain). Samples (20 μg of total protein) were mixed with 4× non reducing loading buffer, heated 15 min at 37°C and subjected to 9% SDS-PAGE gel with 0.1% gelatin (w/v) (Sigma, Spain). Gels were washed twice for 30 min at room temperature with slight shaking in enzymatic activation buffer (50 mM Tris-HCl, 6 mM CaCl_2_, 1.5 μM ZnCl_2_, pH 7.4) with 2.5% Tween-20 to remove SDS and restore gelatinase activity. Gels were then incubated at 37°C for 48 h in enzymatic activation buffer without Tween-20. Gels were stained in Coomasie brilliant blue (Bio-Rad, Spain) until gels were dark blue colored, followed by immersion in discoloring solution (40% methanol, 10% acetic acid) until bands were clear. Gels were digitized and bands were quantified by ImageJ Software version 1.42q (NIH, Bethesda, MD, USA).

### Gel Zymography of Tissue Plasminogen Activator (tPA) Activity

Tissue plasminogen activator (tPA) was measured because of its role on MPP-9 proteolytic activation. tPA activity was assessed in the same samples mentioned above. Samples were mixed with 4× non reducing loading buffer, heated 15 min at 37°C and subjected to 9% SDS-PAGE gel with 0.1% plasminogen (Millipore, Spain) and 0.1% casein (Sigma-Aldrich, Spain). Gels were washed twice for 30 min at room temperature with slight shaking in modified enzymatic activation buffer (0.1 M Tris-HCl, 0.1 M glycine, 0.01 M EDTA, pH 8.0) with 2.5% Tween-20 to remove SDS and restore gelatinase activity. Gels were then incubated at 37°C for 48 h in modified enzymatic activation buffer without Tween-20. tPA activity bands were developed and quantified in the same way that we mentioned above.

### Western Blotting

To measure levels of GA and MMP-9 proteins in each brain region, Western blotting was performed with purified specific polyclonal antibodies against KGA and GAB, as well as commercial antibodies against MMP-9 (Millipore, Spain AB19016, 1/1000). Protein samples (40 μg for KGA and GAB or 20 μg for MMP-9 detection) were separated on SDS-PAGE gels and transferred to nitrocellulose or PVDF membranes. After blocking at room temperature for 1 h with 5% bovine serum albumin (BSA) in TBST buffer (0.1% Tween 20 in Tris-buffered saline, TBS, 100 mM Tris, 150 mM NaCl, pH 7.5), membranes were incubated with primary antibodies in blocking buffer with 5% BSA, overnight at 4°C. After incubation with secondary antibody, the blots were developed by enhanced chemiluminescense technique as recommended by the supplier (Pierce) and bands quantified using the Chemi Doc System (BioRad). β-actin was quantified and used as a loading control.

### Statistics

All statistical analyses were conducted using two-tailed unpaired *t*-tests (WT vs. KOLPA_1_) except when the assumptions of normality and/or homoscedasticicity were violated (Golgi staining). In the latter case, non-parametric Mann-Whitney tests were carried out to compare WT and KOLPA_1_ genotypes. The significance level was set at 5%. GraphPad Prism 7 software (La Jolla, CA, USA) was used to perform all statistical analyses.

## Results

### Silencing of LPA_1_ Sharply Decreases KGA, but Not GLS2, Protein Expression

**Figure 1:**
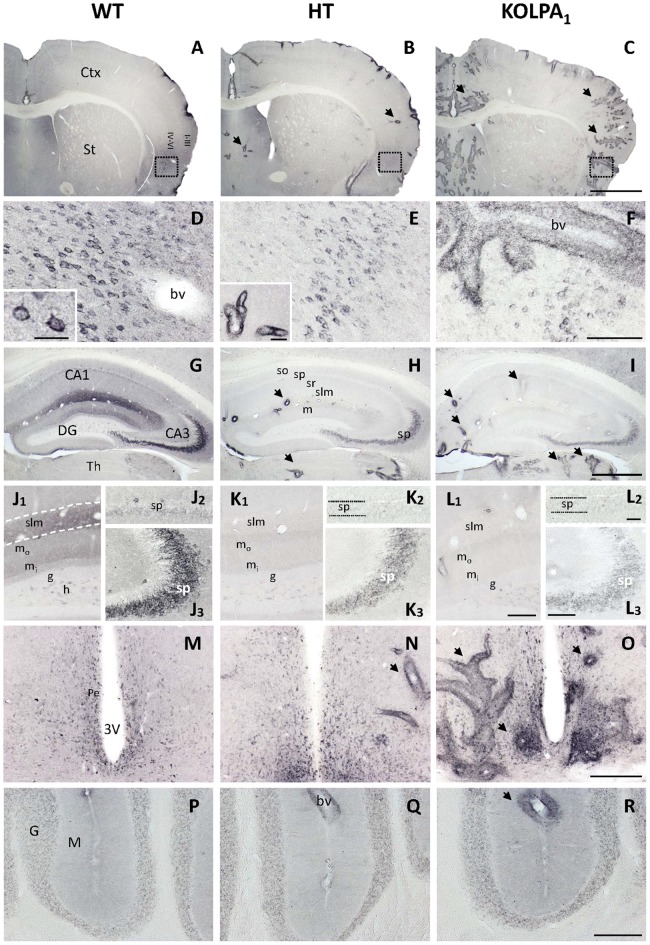
Comparative analysis of immunostaining for Gls-encoded long glutaminase protein variant (KGA) in wild-type (WT), heterozygous (HT) and KOLPA_1_ mice brain. Specific immunolabeling for KGA was detected within the somata/neuropile of several brain regions in WT brain (left column). In general, there was a strong reduction in the staining together with a progressive switch to a perivascular location from control to HT and KO mice **(A–C)**, panoramic views of sections containing cerebral cortex and striatum, black arrows indicate KGA-positive blood vessels). **(D–F)** Progressive change of KGA-staining in deep layers of agranular insular cortex (square; detail of cortical somatic staining, inset in **(D)**; detail of blood vessel, inset in **E**). **(G–I)** KGA-positive staining in the CA1-CA3 hippocampal subfields and DG was dramatically decreased in both HT and KO genotypes in comparison to WT. **(J–L)** DG and *slm* neuropile stainings were found to be very affected in HT **(K1)** and KOLPA1 **(L1)** mice in comparison to WT **(J1)**, as well as CA3 principal neurons **(J2–L2)**, and to a lesser extent equivalent CA1 neurons **(J3–L3)**. **(M–O)** Ventrally, in the hypothalamic levels, we found good examples of this gradual change to perivascular staining around the 3V. No differences were detected among the different genotypes in cerebellum **(P–R)**. Ctx, cortex; St, striatum; bv, blood vessel; CA1, CA3, hippocampal subfields; DG, dentate gyrus; Th, thalamus; so, stratum oriens; sp, stratum pyramidale; sr, stratum radiatum; slm, stratum lacunosum-moleculare; m, molecular layer; m_o_, outer molecular layer; m_i_, inner molecular layer; h, hilus; g, granular layer; Pe, periventricular hypothalamic nucleus; 3V, third ventricle; G, cerebelar granular layer; M, cerebelar molecular layer. Scale bar: **A–C**, 1 mm; **D–F**, 100 μm; **G–I**, 500 μm; **J1–L1**, 50 μm; **J2–L2** and **J3–L3**, 20 μm; **M–R**, 200 μm; inset: **D**, 25 μm; inset: **E**, 40 μm.

Four different GA isoenzymes are expressed in mammalian brain (Márquez et al., 2016): KGA and Gls-encoded short glutaminase protein variant (GAC) proteins are encoded by the *Gls* gene (GLS isoforms), whilst GAB and Gls2-encoded short glutaminase protein variant (LGA) arise from the *Gls2* gene (GLS2 isoforms; Aledo et al., 2000; Matés et al., 2013). The influence of silencing LPA_1_ receptor on brain GA protein expression was assessed by immunohistochemistry of the KGA and GLS2 (GAB/LGA) isoforms. The isoform-specific anti-KGA antibodies employed were raised for a unique C-terminal region of this isoform, which is absent in the GAC isoform, and hence they cannot recognize this second GLS isoform (GAC); however, GAB antibodies would recognize both GLS2 isoforms (GAB and LGA). The immunostaining obtained for KGA (Figure 1) revealed a cytoplasmic punctate pattern typical for a mitochondrial localization, in accordance with previous reports on KGA localization in mammalian brain (Olalla et al., 2002; Cardona et al., 2015). However, a drastic decrease in the intensity of the staining was clearly seen in several cerebral regions from HT and KO mice, like cortex (Figures 1B,C, details in Figures 1E,F, respectively) and hippocampus (Figures 1H,I, respectively) in comparison to the same areas in WT mice (Figures 1A,D,G). Specifically, in the hippocampus, CA3 pyramidal layer neurons (Figures 1J3–L3) and the neuropile of both CA1 stratum lacunosum-moleculare and DG molecular layer (Figures 1J1–L1) were among the most affected. In addition, neuronal KGA-immunolabeling in certain cortical regions, like deep layers of agranular insular cortex (Figures 1D–F), was also notably reduced, whereas in others, as the cerebellum, non-specific alteration was observed (Figures 1P–R). Interestingly, these reductions in the KGA labeling were generally gradual from control to HT and KO animals, which seem to reflect a proportional decline on KGA protein expression when LPA_1_ receptor levels were diminished. Also progressive was the appearance of intense perivascular immunoreactivity in animals with decreased LPA_1_ expression, especially in the KO mice (Figures 1C,F,M–O).

**Figure 2:**
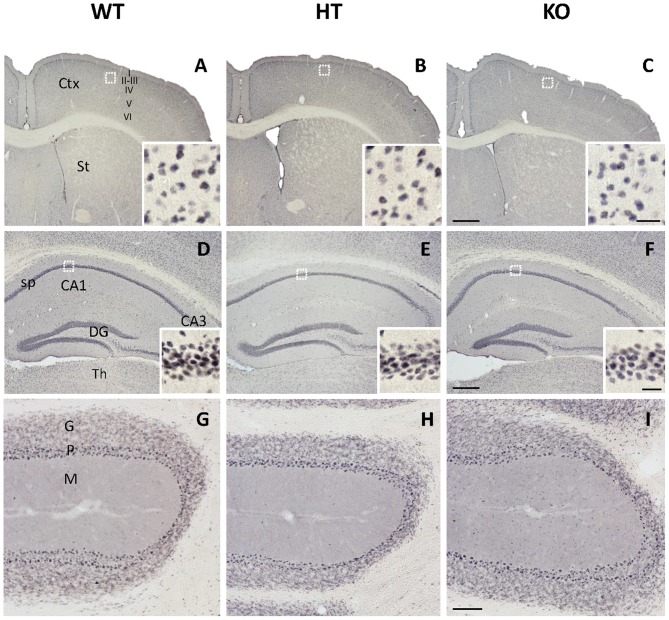
Immunohistochemal staining for glutaminase gene encoding the liver-type isoforms (GLS2) in WT, HT and KOLPA_1_ mice brain. Immunolabeling for GLS2 isoforms yielded a nuclear staining in all the brain regions analyzed by this technique. **(A–C)** Cerebral cortex and insets showing details from layer III of the motor cortex (white squares). **(D–F)** Hippocampus and details from CA1 stratum pyramidale in insets. **(G–I)** Details from cerebellum are depicted. No differences were detected among the different genotypes in any of these cerebral areas. Ctx, cortex; St, striatum; CA1, CA3, hippocampal subfields; sp, stratum pyramidale; DG, dentate gyrus; Th, thalamus; G, cerebelar granular layer; P, Purkinje layer; M, cerebelar molecular layer. Scale bar: **A–C**: 500 μm, **D–F**: 250 μm, **G–I**: 100 μm; Insets: 20 μm.

**Figure 3:**
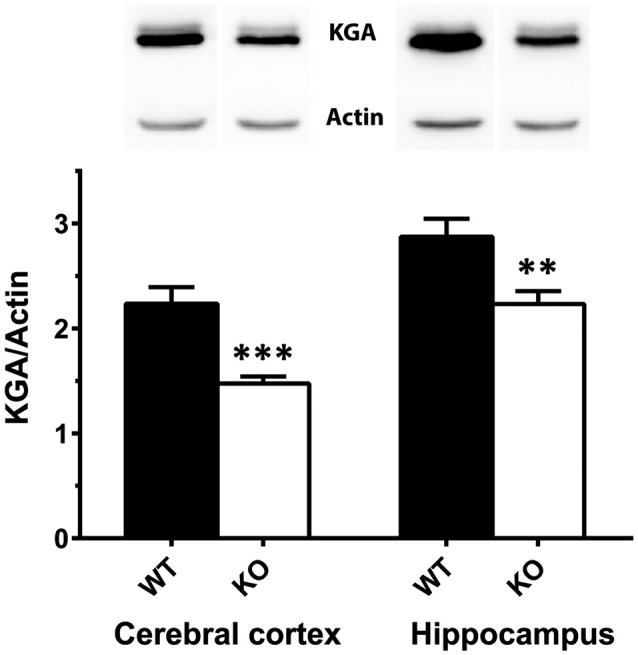
Immunoblot analysis of KGA protein expression in cerebral cortex and hippocampus. Total protein extracts (40 μg) were isolated from cerebral cortex and hippocampus of WT and KOLPA_1_ mice and analyzed by Western blotting using isoform-specific KGA antibodies. A representative Western blot is shown in the top panel, using β-actin as a loading control. Densitometry analysis of the blots was done using the Chemi Doc System (Bio-Rad). KGA expression values normalized to β-actin are shown in the bottom panel. Values are means ± SEM of nine animals of each phenotype (WT and KOLPA_1_). ***p < 0.001; **p < 0.01.

In contrast with KGA, the immunolabeling patterns against GLS2 isoforms were roughly similar for the three types of mouse strains studied (Figure 2). No apparent change on GLS2 expression was found in the different brain regions analyzed by immunohistochemistry such as brain cortex, striatum (Figures 2A–C), hippocampus (Figures 2D–F) or cerebellum (Figures 2G–I). As expected, the appearance of the immunolabeling revealed a distinct pattern compared with KGA, showing a broad nuclear staining characteristic of GLS2 in mammalian brain (insets in Figure 2), in agreement with previously reported data (Olalla et al., 2002; Cardona et al., 2015). Finally, supporting experimental evidences were obtained by immunoblot analysis of total protein extracts isolated from whole cerebral cortex and hippocampus and revealed with isoform-specific KGA antibodies (Figure 3). In agreement with immunohistochemical data revealing a decreased KGA protein expression, Western blot results also showed a significant reduction of KGA protein in both regions (Figure 3). Meanwhile, GLS2 protein levels were not significantly altered in cerebral cortex and hippocampus as assessed by immunoblot assays (results not shown).

### Enzymatic Activity and GLS mRNA Levels in KOLPA_1_ Mice

**Figure 4:**
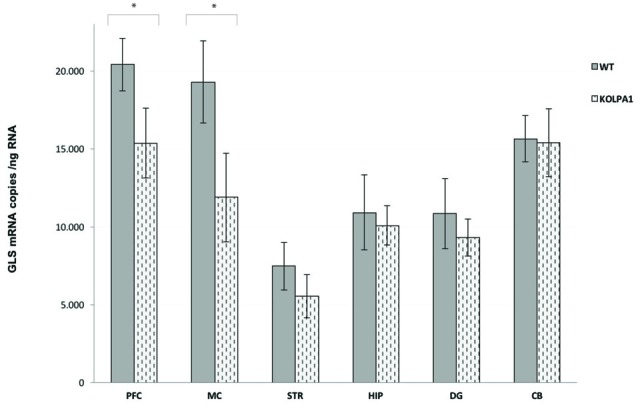
Quantitative measurement of GLS mRNA levels by real-time RT-PCR. Total RNA was isolated from the different brain regions and absolute quantifications of GLS mRNA levels were performed as described in the Methods section. Five animals of each genotype were analyzed. The GLS transcripts are expressed as numbers of molecules of mRNA per ng of total RNA ± SEM (five independent experiments done in triplicate). *p < 0.05. PFC, prefrontal cortex; MC, motor cortex; STR, striatum; HIP, hippocampus; DG, dentate gyrus; CB, cerebellum.

**Figure 5:**
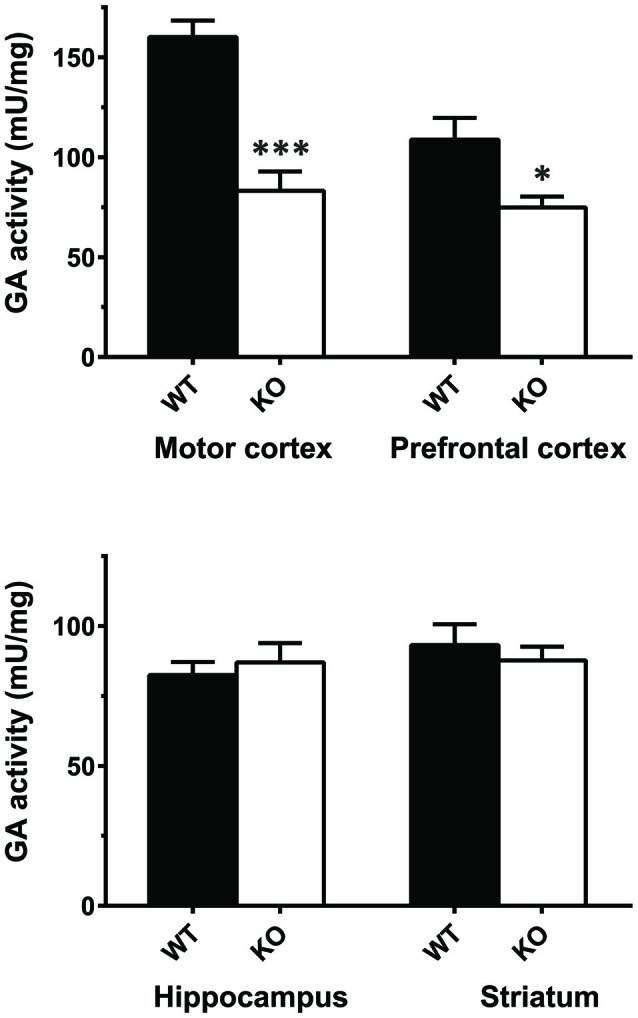
Determination of total glutaminase (GA) activity in mouse brain regions. Significant differences in GA specific activity were found in motor cortex (WT *n* = 9; KO *n* = 5) and PFC (WT *n* = 12; KO *n* = 8), but not in hippocampus (WT *n* = 16; KO *n* = 12) and striatum (WT *n* = 15; KO *n* = 8). ***p < 0.001; *p < 0.05.

The results of real-time PCR indicate that mRNA levels of GLS (KGA + GAC) remained essentially unchanged in KOLPA_1_ mice, with the exception of prefrontal and motor cortex where a significant decrease was observed (Figure 4; see also Castilla-Ortega et al., 2016). With regard to GA activity, we assayed enzyme catalytic activity in total protein extracts isolated from individual brain areas. The GA activity measured in whole regions from WT and KOLPA_1_ mice did not show significant variations, except for the motor cortex and PFC of LPA_1_-deficient mice which showed a significant bias to lower activity levels (Figure 5). Altogether, the results suggest a lack of correlation between GLS mRNA levels and protein/activity values in most cerebral regions studied, except for two cerebral cortex subregions: motor and PFC, where diminished mRNA levels correlated with reduced KGA protein and GA activity values.

### Anomalous Dendritic Spine Morphology in KOLPA_1_ in Mice

**Figure 6:**
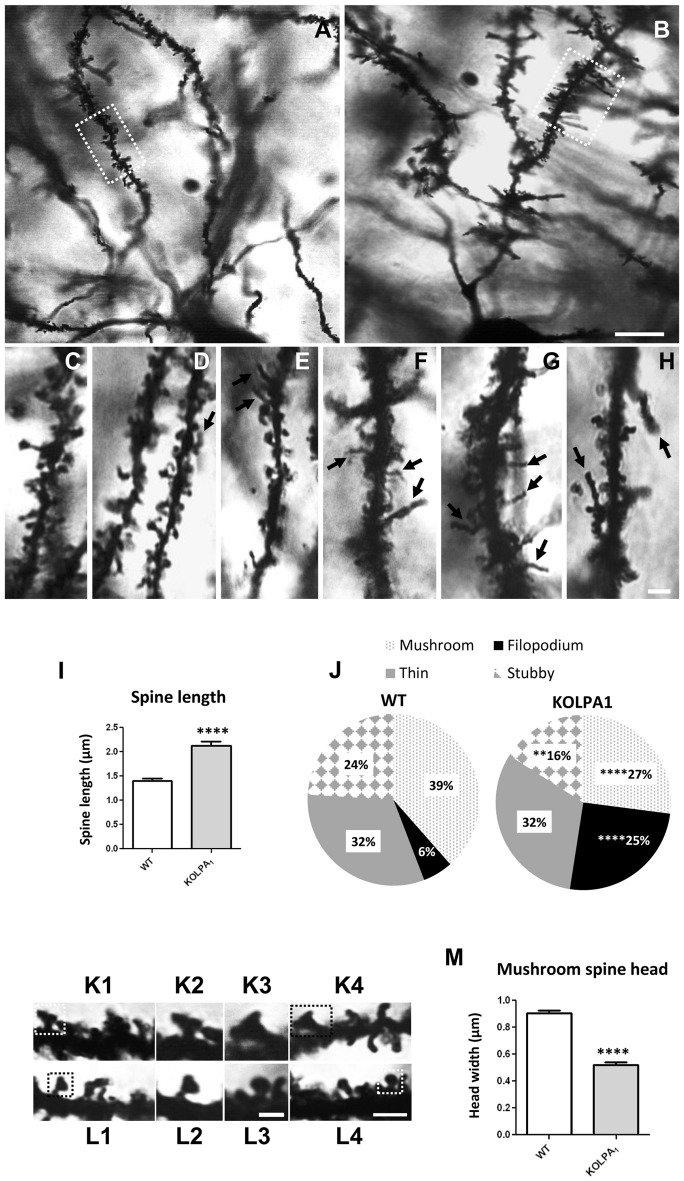
Golgi-Cox impregnated basal tufts of CA1 pyramidal neurons from WT **(A)** and KOLPA_1_ **(B)** mice. Segments from basal dendrites (stratum oriens, white rectangles in **(A,B)** highlight representative dendritic segments) demonstrating the morphological differences between KOLPA_1_ **(F–H)** and WT **(C–E)** spines. Black arrows point to dendritic filopodia. **(I)** Overall spine length values showed significant differences between genotypes (WT 1.4 ± 0.05 μm, KOLPA_1_ 2.12 ± 0.09 μm; Mann-Whitney, ****p < 0.0001). **(J)** Significant differences were found in the distribution of dendritic spine morphologies within stratum oriens from KOLPA_1_ model compared to the same region of WT mice. Filopodia type was significantly more frequent in KOLPA_1_ mice in detriment of both mushroom and stubby categories (*t*-test, Filopodia ****p < 0.0001, Stubby **p = 0.0073 and Thin, n.s.; Mann-Whitney, Mushroom ****p < 0.0001). **(K,L)** Spine head of mushroom subtype was smaller in KOLPA_1_ (**L**; **L2,L3** depict details from **L1** and **L4**, respectively) than in WT mice (**K**; **K2,K3** depict details from **K1** and **K4**, respectively). **(M)** Quantitative analysis demonstrated a significant decrease in the head-width of mushroom spines from KOLPA_1_ mice (0.52 ± 0.02 μm) compared to WT (0.91 ± 0.02 μm); *t*-test, ****p < 0.0001. Scale bar: **A,B** (10 μm); **C–H** (2 μm); **K1,K4,L1** and **L4** (2 μm); **K2,K3,L2** and **L3** (1 μm).

The potential involvement of LPA signaling in developmental process and neurotransmitter handling has been previously reported. Glutamate has been also involved in trophic functions and neuronal development. Therefore, we wanted to check whether the absence of LPA_1_ receptor and/or the strong deficit observed in GLS protein expression would affect the dendritic spines. Since hippocampus exhibited a pronounced KGA deficit and the behavioral phenotype of the LPA_1_ deficient mice are mainly attributed to hippocampal malfunction (Santin et al., 2009; Castilla-Ortega et al., 2010; Blanco et al., 2012a), we decided to assess dendritic spine density and morphology using the Golgi-Cox staining in the CA1 region. Representative images of basal pyramidal dendrites in CA1 are shown in Figure 6A (WT) and Figure 6B (KOLPA_1_). First, we quantified the spine density (number of spines per 10-μm long dendritic segments) in nonprimary dendrites. Although we did not find statistically significant differences (not shown) neither in spine density nor dendrite diameter (Mann-Whitney, *p* = 0.12 and *p* = 0.86, respectively), between KOLPA_1_ and WT mice, microscopic analysis revealed morphological alterations in the KO dendritic spines. Subsequently, we performed a detailed morphometric analysis of CA1 pyramidal dendrites, and first observed that dendritic spines from this region were longer, tortuous and thinner in KO (Figures 6F–H) than in WT mice (Figures 6C–E), exhibiting the typical characteristics of immature states (Ebrahimi and Okabe, 2014). Overall spine length values evidenced significant differences between genotypes in this parameter: a clear trend was found towards longer spines in the KOLPA_1_ mice (Figure 6I). Then, comparing lengths between the subtypes of spines (Table 1 below), we also found significant differences in thin spines between genotypes. Moreover, quantitative analysis of spine types (mushroom, thin, stubby or filopodia) indicated drastic changes in spine populations: filopodia was significantly more frequent in KO mice in detriment of the mushroom and stubby categories, which showed marked decreases in the null LPA1 mice (Figure 6J). In addition, the spine head-width of mushroom subtype was significant smaller in KOLPA_1_ spines (Figure 6L) than in WT (Figure 6K; *t*-test, ****p < 0.0001; Figure 6M).

**Table 1:** Length (mean ± SEM; μm) of dendritic spine subtypes analyzed from wild-type (WT) and KOLPA_1_ mice.

Subtype	WT	KOLPA_1_
Mushroom	1.24 ± 0.04	1.26 ± 0.05
Thin***	1.61 ± 0.07	1.95 ± 0.07
Stubby	0.71 ± 0.02	0.65 ± 0.03
Filopodia	4.2 ± 0.3	4.2 ± 0.2

***p = 0.0002 (Mann-Whitney).

Therefore, these results strongly suggest that the formation/maturation of dendritic spines could be significantly impaired in the hippocampal pyramidal neurons of KOLPA_1_ mice.

### Silencing of LPA_1_ Reduces MMP-9, but Not MMP-2 and tPA Activities

**Figure 7:**
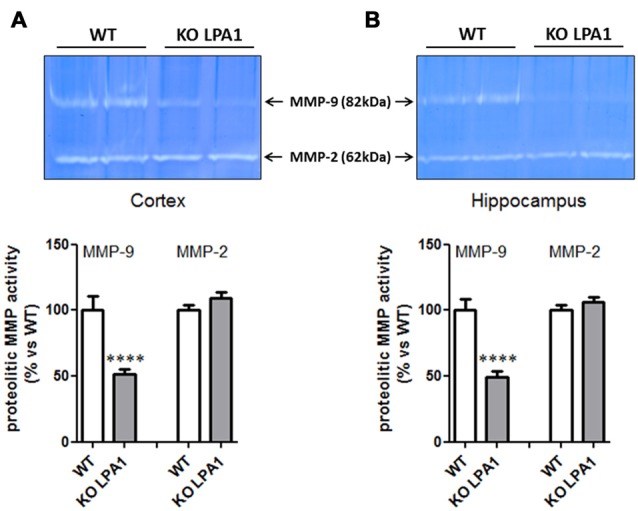
Zymograms of cortical **(A)** and hippocampal **(B)** gelatinase activity from WT and KOLPA1 mice. Matrix metalloproteinase (MMP)-9 and MMP-2 activities were determined in hippocampus and cerebral cortex (whole hemisphere) from both genotypes (WT *n* = 8; KO *n* = 11). Significant differences were found in MMP-9 proteolytic activity of both brain areas, showing a decreased activity in KO mice (****p < 0.0001), while MMP-2 remained unchanged.

To identify molecular candidates which entrain this structural change, we investigated alterations in protein expression profiles of dendritic markers known to be involved in the formation and modulation of the dendritic spine synapse. Immunoblot analysis of post-synaptic density protein 95 (PSD95) and synaptophysin revealed no significant changes in their expression levels between WT and mutant KOLPA_1_ mice (results not shown). Matrix metalloproteinases (MMP) have attracted attention recently as important modulators of spine morphology, along with other constitutive proteins well known to be involved in synapsis formation. Several of these proteins were analyzed in order to search for altered expression patterns which might underlie some of the changes observed in spine structure. Gelatin zymography reveals a band at approximately 82 kDa corresponding to the active form of MMP-9 (Figure 7). Quantitative image analysis revealed a significant decrease on MMP-9 activity in cortex (Figure 7A; *p* = 0.0001) and hippocampus (Figure 7B; *p* < 0.0001) of KOLPA_1_ mice. The overall decrease observed was around a 50% in the LPA_1_-deficient mice. There was no change in MMP-2 activity (cortex, *p* = 0.16; hippocampus, *p* = 0.26).

**Figure 8:**
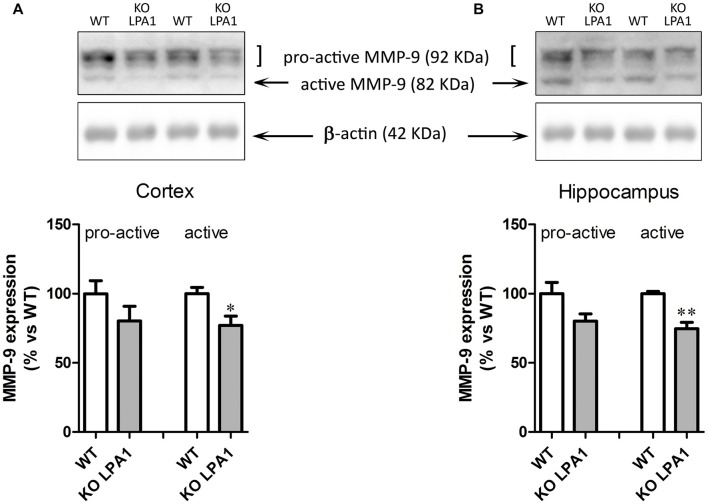
Immunoblot analysis of MMP-9 protein expression in cerebral cortex **(A)** and hippocampus **(B)** from WT and KOLPA_1_ mice. Quantitive analysis of proactive (92 kDa) and active (82 kDa) MMP-9 were determined by densitometry and relative to β-actin expression (WT *n* = 4; KO *n* = 5). A reduction in active MMP-9 expression were detected in KOLPA_1_ compared to WT mice (*p < 0.05, **p < 0.01), while no changes were detected in pro-active MMP-9 expression.

To study the possibility that reduced MMP-9 activity was due to diminished enzyme expression, Western blot analysis was performed. Pro-active and active MMP-9 bands were detected at 92 and 82 kDa, respectively (Figure 8). Quantitative image analysis of each of the two bands revealed a reduction in active MMP-9 (Figure 8, cortex, *p* = 0.03; hippocampus, *p* = 0.0025) of KOLPA1 mice. No changes were detected in pro-active MMP-9 brain expression (Figure 8, cortex, *p* = 0.22; hippocampus, *p* = 0.07).

**Figure 9:**
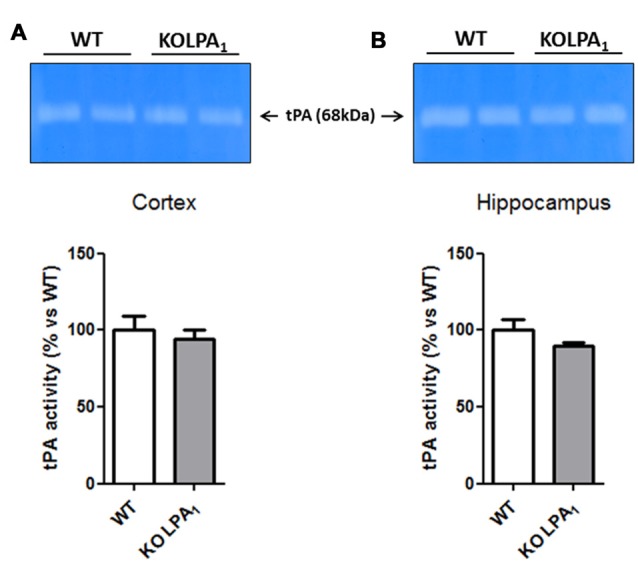
Zymograms of tissue plasminogen activator (tPA) activity in the cortex **(A)** and hippocampus **(B)** of WT and KOLPA_1_ mice. Quantitative analysis of tPA activity was determined by densitometry of the 68 kDa band of substrate degradation (WT *n* = 4; KO *n* = 6). No changes in tPA activity were detected between both type of mice.

Although tPA/plasmin system has been involved in numerous aspects of brain function, including dendritic remodeling in mouse hippocampus (Pawlak et al., 2005) and visual cortex (Mataga et al., 2004), as well as inducing MMP-9 proteolytic activation since pro-active form (Wang et al., 2003), we did not detect any significant changes in tPA activity in cortex (Figure 9A, *p* = 0.60) and hippocampus (Figure 9B, *p* = 0.16) of KOLPA_1_ compared with WT mice.

## Discussion

### LPA_1_ Deficit Decreases KGA Protein Expression in Key Glutamatergic Regions

Using affinity-purified isoform-specific antibodies and immunohistochemistry we revealed a marked deficit of the most abundant cerebral GA: the KGA isoform encoded by the *Gls* gene (Blanco et al., 2012b; Martín-Rufián et al., 2012). This drastic decrease of KGA protein expression, particularly relevant in cerebral cortex and hippocampus, was completely unexpected and cannot be explained by previous experimental data reporting alterations in the brain of this KO mouse model. Although a decrease of adult cortical neurons (due to an increased cortical cell death) was previously described in KOLPA_1_ mice (Estivill-Torrús et al., 2008), the reported lower number of cortical neurons only occurs in layers II/III, V and VI and seems insufficient to completely justify the strong reduction found for KGA protein in cerebral cortex. Similarly, the reduced neurogenesis in the mouse DG previously noted for KOLPA_1_ mice (Matas-Rico et al., 2008) may contribute to part of the deficit shown by KGA in this subregion, but it does not explain the dramatic silencing of KGA expression in other hippocampal regions. Despite the overall fall in KGA protein levels, one striking feature observed was the intense perivascular mark, which positively correlated with LPA_1_ silencing. This gradual shift of KGA staining toward regions surrounding blood vessels may suggest a mechanism to control vascular permeability in LPA_1_-deficient mice. It is well known that LPA is a critical factor for regulating angiogenesis in vertebrates (Yukiura et al., 2011), as well as tight junction permeability in cultured endothelial cells (Schulze et al., 1997). Therefore, HT and KO animals may have defects on brain vascular development and/or blood-brain barrier permeability. Interestingly, glutamate is a vasoactive compound and glutamate receptors are expressed in perivascular glia and vascular endothelial cells; hence, the intense vascular KGA reactivity would be a functional response aimed at regulating the vascular tone under pathophysiological conditions (reduced or null LPA_1_ expression). Of note, a role of GA in cerebrovascular regulation was first postulated by our group after demonstrating concrete perivascular locations for brain GA isoforms (Olalla et al., 2008; Márquez et al., 2009).

Correlation between GLS mRNA abundance, KGA protein levels and GA activity was only apparent in two brain regions: prefrontal and motor cortex, which showed significant decreases at the three levels examined. Mice lacking LPA_1_ exhibit motor alterations; thus, reductions in locomotor activity, hypolocomotion during first exposure to the open field and reduced exploratory reactivity to a novel environment have been previously reported (Harrison et al., 2003; Santin et al., 2009). In contrast, the GLS mRNA levels in other brain areas, like hippocampus, remained essentially unaltered; therefore, transcript abundance did not parallel the behavior shown by KGA protein levels. This dissociation between transcript and protein levels for cerebral GA isoforms was previously found in studies dealing with their involvement in the addictive behavior of cocaine abuse (Blanco et al., 2012b) or in neuronal differentiation (Agostini et al., 2016). Hence, the mechanism(s) underlying the huge decline of KGA protein levels in brain hippocampus and cerebral cortex regions of KOLPA_1_ mice, other than motor and PFC, should be mostly posttranscriptional.

The maintenance of GA activity in key glutamatergic regions of KOLPA_1_ mice, despite substantial decrements in KGA protein, immediately suggests the existence of compensatory mechanisms. Accordingly, the glutamate content in PFC and hippocampus of KOLPA_1_ mice did not change as compared with WT mice (Harrison et al., 2003), reinforcing the view that other GA isozymes may be activated to keep constant glutamate levels. Although the contribution of each GA isoform to the pool of neurotransmitter Glu is currently unknown, KGA is the most abundant GA in mammalian brain (Olalla et al., 2002; Martín-Rufián et al., 2012) and the principal source of neurotransmitter glutamate (Márquez et al., 2016 and references therein). Thereby, a plausible explanation is that neurons attempt to compensate for the loss of KGA protein by increasing the process of glutamate generation through other GA isoforms expressed in brain (e.g., enhanced local activity of GLS2 isozymes). Indeed, two distinct LPA-related mechanisms are known to enhance the activity of GLS isoforms and might be operative here via other LPARs distinct from LPA_1_. The alternative spliced isoform of KGA, named GAC, is activated by Rho GTPases (Wang et al., 2010); interestingly, LPA stimulates activation of Rho in several neuronal cell lines, cortical progenitors and cortical neurons (Ye et al., 2002). Also, the Raf-Mek-Erk signaling pathway has been shown to increase KGA activity by phosphorylation after stimulation by several mitogens, including LPA (Thangavelu et al., 2012); therefore, post-translational modifications of the remaining KGA protein pool would cooperate in keeping basal GA activity in mutant mice. On the other hand, the existence of compensatory processes affecting glutamate signaling enzymes and receptors in KO mouse models has been previously reported. For example, null mice for the dopamine D_4_ receptor displayed enhanced expression of dopamine D_1_ and glutamate NMDA receptors (Gan et al., 2004), while mice lacking the mGlu1 receptor showed a compensatory overexpression of functional mGlu5 (Rossi et al., 2013). Finally, it is worth mentioning that null mutants mice lacking *Gls* gene –and therefore without KGA protein- died in the first postnatal day (Masson et al., 2006). Nevertheless, the synaptic terminals of *Gls*^−/−^ neurons showed apparently normal glutamate content on individual presynaptic terminals; this unexpected result was explained by compensatory mechanisms of glutamate synthesis (Masson et al., 2006).

### Role of Glutaminases in Neuronal Development and Differentiation

Axonal growth, neurite outgrowth and dendrite remodeling have been described in studies dealing with the role of LPA in developmental processes of the nervous system (Fujiwara et al., 2003). Although the post-transcriptional mechanisms underlying down-regulation of brain KGA after LPA_1_ silencing are currently unknown, the shortage in KGA protein may also give rise to developmental defects in cortical and hippocampal neurons which, in turn, may contribute to strong deficits in neurite outgrowth, axon growth and maturation of dendritic spines. Accordingly, in the KOGLS mouse model, which shows a slight effect on glutamatergic synaptic transmission, the authors concluded that KGA deficit should principally impact the implementation of more active neural network circuits, stressing the importance of KGA in neuronal maturation, development and differentiation (Masson et al., 2006). In fact, KGA has been associated with neuronal differentiation and proliferation of neural cells. Thus, both GLS isoforms (KGA and GAC) were upregulated during neurogenesis of human neural progenitor cells (NPCs), and their expression pattern positively correlated with the neuronal marker microtubule associated protein 2 (MAP-2; Wang et al., 2014). Most important, studies of cultured human NPCs after siRNA silencing of GLS suggest a critical role of GLS isoforms for proliferation and survival of NPCs (Wang et al., 2014). Furthermore, inhibition of GA activity in cultures of mouse embryonary cortical neurons resulted in impaired neuritogenesis (Velletri et al., 2013), while both mRNA and protein KGA levels strongly increased when cerebellar granule cells differentiate in culture (Thomas et al., 1989). This last study also demonstrated KGA upregulation in parallel with Ca^2+^-dependent glutamate release and formation of neurites, synaptic vesicles and synapses (Thomas et al., 1989).

In recent years, the involvement of KGA in the metabolic reprogramming underlying neuronal differentiation has been revealed. Thus, during *ex vivo* differentiation of primary cortical neurons, the glutamate-glutamine pathway was notably increased in parallel with upregulation of the GLS and GLS2 mRNA levels (Agostini et al., 2016). Strikingly, novel neuronal locations, distinct from mitochondria, were recently discovered for KGA through interactions with new scaffolding proteins. Thus, KGA relocalized from neuronal cell body mitochondria to neurite terminals after interaction with the protein BNIP-H (Caytaxin), which is highly expressed at the hippocampus and cerebellum; this specific KGA targeting occurs in PC12 cells when they differentiated to a neuronal phenotype, supporting a role in neuronal cell growth (Buschdorf et al., 2006). A second KGA interacting-partner was later discovered in brain: Bmcc1s, a protein also involved in the regulation of cell morphology (Boulay et al., 2013). In mouse cortical neurons, Bmcc1s interacts with the precursor KGA protein avoiding its proper targeting to the mitochondria and leading to its accumulation within the cytoplasm. Thus, KGA may traffic in neurons to distant sites and is not exclusively confined to the mitochondria; instead of it, KGA seems to be needed for neurite outgrowth, dendrites formation/maturation and establishment of novel synapses. Therefore, it is tempting to speculate that an impairment of KGA targeting to synaptic regions, as expected from the drastic downregulation of KGA shown by KOLPA_1_ mice, would have a negative impact in these mutant mice during brain development.

### Regulation of Dendritic Structure by LPA

It is well documented the role of LPA in synapse formation through modulation of dendritic spine dynamics. For example, in cultured hippocampal neurons, the LPA_1_ receptor was localized in a punctate manner in dendritic spines and its overexpression correlated with increases in the density and size of spines (Pilpel and Segal, 2006). Therefore, we hypothesized that KOLPA_1_ mice would display changes in the morphology of their excitatory synaptic spines. Golgi staining showed hippocampal dendritic spines with a clear immature phenotype in KOLPA_1_ mice: the predominance of filopodia, in detriment of the mushroom and stubby spines, and the marked reduction in mushroom spine head-width are relevant structural changes with potential repercussion in synaptic neurotransmission. Of note, during the first few weeks of postnatal life, immature hippocampal dendrites show numerous filopodia while, in contrast, they are rarely observed in the mature hippocampus, although blocking synaptic transmission triggers again filopodia formation (for a review see Bourne and Harris, 2008).

On the other hand, a considerable body of evidence has recently shown a key role for MMP-9, and other extracellular proteases such as tPA, in synaptic remodeling, synaptic plasticity and cognitive processes (Mataga et al., 2004; Rivera et al., 2010; Michaluk et al., 2011). In neurons, MMP-9 is located at the postsynaptic domains of excitatory synapses and its inhibition has been linked to memory deficits in behavioral learning paradigms (Nagy et al., 2007). Of note, MMP-9 is a key regulator of dendritic spine morphology (Michaluk et al., 2011; Dziembowska and Wlodarczyk, 2012). We therefore examined the effects of LPA_1_ depletion on the activity of three extracellular matrix proteases in hippocampus and cortex: MMP-9, MMP-2 and tPA. Zymography revealed a marked reduction in MMP-9 activity in hippocampus and cortex of KOLPA_1_ mice compared with WT counterparts, without significant changes in MMP-2 nor tPA activities.

MMP-9 activation is strictly regulated during spine maturation (Tian et al., 2007). This inducible MMP is extracellularly secreted in the pro-active form in which catalytic site is blocked by a signal peptide. Several factors induce its activation from pro-active form through the cleavage of the propeptide. Western blot analysis indicates that brain active MMP-9 protein expression is reduced in KOLPA_1_ respect to WT mice, although there is no significant differences in the expression of brain pro-active MMP-9. These results indicate that MMP-9 activity decrement arises due to a decrease in active MMP-9 processing and release by post-translational mechanisms. In sharp contrast, no relevant changes were found for MMP-2 protein in our LPA_1_-deficient animal models. Remarkably, in animal models of drug addiction an activation of MMP-2 occurs after extended withdrawal from drug use: increases in MMP-2 activity, along with enhanced spine head diameter and spine number, are persistent neuroadaptations that occur after chronic drug exposure and underpin the enduring vulnerability to drug relapse (Mulholland et al., 2016).

The marked reduction of MMP-9 activity in KOLPA_1_ mice would prevent transient synaptic plasticity changes needed for synaptic strength, due to the lack of adequate re-shaping of spines in hippocampal neurons, thereby having important functional correlates in the neurophysiological phenotype of these animals. Interestingly, MMP-9 activity promoted maturation of dendritic spines and drive spine enlargement and synaptic potentiation (Wang et al., 2008). Moreover, spines undergo actin-dependent increases in spine-head volume during hippocampal long-term potentiation (LTP), whilst the levels of both the precursor and the active form of MMP-9 are notably elevated in the CA1 region, suggesting that upregulation of MMP-9 is related mechanistically to LTP (Huntley, 2012 and references therein). In addition, MMP-9 needs to be expressed at very high levels in the postnatal hippocampus and regulation of its expression control neuron numbers (Murase and McKay, 2012). Thus, the downregulation of MMP-9 activity shown by LPA_1_ null mice may play an important role in their cognitive performance: the lack of synaptic plasticity induced by MMP-9-dependent hippocampal spine remodeling would partially explain the cognitive and memory deficits reported in these animals. The reduced MMP-9 activity would be a downstream effect of the absence of LPA-induced Rho/Rho kinase (ROCK) signaling pathway, which is known to produce proteolytic enzymes like MMP-9 (Yu et al., 2014). Nevertheless, other authors found that spine morphology is affected by LPA_1_ independently of its three main signaling cascades; therefore, they suggest this effect was mostly mediated by interaction with other synaptic proteins (LPA_1_ has a PDZ-binding module at its C-terminal region; Pilpel and Segal, 2006).

## Conclusion

Our results suggest that LPA_1_ exerts an important impact on KGA expression mainly through a posttranscriptional mechanism, stressing a key relationship between neuroactive lipids as LPA and glutamatergic transmission. Furthermore, the absence of LPA_1_ signaling downregulates expression of active MMP-9 and provokes drastic changes in hippocampal dendritic spines toward an immature phenotype. Taken together, these changes may provide a molecular basis for the role of LPA in influencing synaptic plasticity associated to cognitive and memory processes. Further studies are needed to address the concrete mechanism linking LPA_1_ signaling and GA expression in key glutamatergic regions and its relevance for cognitive function.

## Author Contributions

AG, MIC, FRF and JM: conceived and designed the experiments. JAC-S, AP, RS-V and CC: immunohistochemistry. JAC-S, LC and AP: western blots and activity measurements. MM-R and FJA: real-time RT-PCR. GE-T, EB and FRF: maintenance, selection and supply of KO mice and brain tissues. AP and EB: quantification and statistical analysis. AP, JAC-S and RS-V: golgi staining. MP-H and MIC: metalloproteinases and tPA assays. AG, FRF and JM: analyzed the data. JM: wrote the article.

## Conflict of Interest Statement

The authors declare that the research was conducted in the absence of any commercial or financial relationships that could be construed as a potential conflict of interest.

## Abbreviations

DAB: diaminobenzidine tetrahydrochloride
GA: glutaminase
GAB: *Gls2*-encoded long glutaminase protein variant
GAC: *Gls*-encoded short glutaminase protein variant
*Gls*: glutaminase gene encoding the kidney-type isoforms
*Gls2*: glutaminase gene encoding the liver-type isoforms
KGA: *Gls*-encoded long glutaminase protein variant
LGA: *Gls2*-encoded short glutaminase protein variant
LPA: lysophosphatidic acid
LPARs: lysophosphatidic acid receptors
LTP: long-term potentiation
MMP: matrix metalloproteinase
NMDA: N-methyl-D-aspartate
NPCs: neural progenitor cells
NP-40: Nonidet-P40
PBS: phosphate-buffered saline
PFC: prefrontal cortex
PSD95: post-synaptic density protein 95
ROCK: Rho kinase
TBS: Tris-buffered saline
TCA: trichloroacetic acid
tPA: tissue plasminogen activator.
